# Floating Matrix Dosage Form for Propranolol Hydrochloride Based on Gas Formation Technique: Development and *In Vitro* Evaluation

**DOI:** 10.3797/scipharm.0909-02

**Published:** 2010-09-26

**Authors:** Kiran Chaturvedi, S. Umadevi, Subhash Vaghani

**Affiliations:** 1 P. E. S. College of Pharmacy, Bangalore, India; 2 Smt. R. B. Patel Mahila Pharmacy College, Atkot, Gujarat, India

**Keywords:** Propranolol hydrochloride, Gastro retentive drug delivery system, Sustained release, Scanning Electron Microscopy (SEM), Gel matrix, X-Ray Powder Diffractometry (XRPD)

## Abstract

Gastroretentive tablets of propranolol hydrochloride were developed by direct compression method using citric acid and sodium bicarbonate as the effervescent base. Hydroxypropyl methylcellulose; HPMC K15M was used to prepare the floating tablets to retard the drug release for 12h in stomach. Na-carboxymethyl cellulose (NaCMC) or carbopol 934P was added to alter the drug release profile or the dimensional stability of the formulation. Dicalcium phosphate (DCP) was used as filler. Formulations were evaluated for floating lag time, duration of floating, dimensional stability, drug content and *in vitro* drug release profile. The formulations were found to have floating lag time less than 1min. It was found that the dimensional stability of the formulations increase with increasing concentration of the swelling agent. The release mechanism of propranolol hydrochloride from floating tablets was evaluated on the basis of Peppas and Higuchi model. The ‘n’ value of the formulations ranged from 0.5201 to 0.7367 (0.5<n<1.0) which indicated anomalous (non-Fickian) transport mechanism. Formulation containing 27.5% HPMC K15M, 29% DCP, 3.75% citric acid and 18.75% sodium bicarbonate seemed most desirable. FTIR, DSC and XRPD studies indicated the absence of any significant chemical interaction within dug and excipients. Stability study of optimized formulation revealed no significant change and found to be stable.

## Introduction

Gastric emptying of dosage forms is an extremely variable process. Short and variable gastric emptying time can result in incomplete drug release from the delivery system above the absorption zone (stomach or upper part of small intestine), leading to diminished efficacy of the administered dose [1]. Gastric retention provides advantages such as the delivery of drugs with narrow absorption windows in the small intestinal region. In addition, longer residence time in the stomach could be advantageous for local action in the upper part of the small intestine, for example treatment of peptic ulcer disease. Furthermore, improved bioavailability is expected for drugs that are readily absorbed upon release in the GI tract. These drugs can be delivered ideally by slow release from the stomach [2]. Various approaches are available for prolonging the residence time of drugs in the GI tract; among which floating drug delivery has drawn considerable attention. The system basically floats in the gastric fluid because of its lower density compared to that of the aqueous medium [3]. Floating oral delivery systems are expected to remain buoyant in a lasting way upon the gastric contents and consequently to enhance the bioavailability of drugs. The lasting intragastric buoyancy of a controlled release dosage form might also provide a suitable manner to constantly deliver a drug locally into the stomach and hence achieve a sustained site-specific therapeutic action.

Mucoadhesive systems have gained considerable attention due to their ability to adhere to the mucus layer, as well as to release the drug in a sustained manner. Propranolol, a non-selective beta adrenergic blocking agent, has been widely used in the treatment of hypertension, angina pectoris, and many other cardiovascular disorders. It is highly lipophilic and is almost completely absorbed after oral administration. However, much of the drug is metabolized by the liver during its first passage through the portal circulation; on average, only about 25% reaches the systemic circulation [4], its elimination half-life is also relatively short (about 2–6 h) [5]. It shows pH dependent solubility; solubility at pH 1.2 is 225 mg/ml, while at pH 6.8 it is 130 mg/ml [6]. In addition, the drug is stable at acidic pH only; decomposes rapidly when alkaline. Solutions are most stable at pH 3; in aqueous solutions, propranolol decomposes with oxidation of the isopropylamine side-chain. Conventional tablets of propranolol hydrochloride contains a dose of 20 to 40 mg and used for 3 to 4 times a day in the management of several cardiovascular disorders such as hypertension, angina pectoris and arrhythmias. Such frequent drug administration may reduce patient compliance and therapeutic efficacy. The aim of the present study was to formulate floating tablets of propranolol hydrochloride based on gas formation technique in order to prolong its stay in the gastric medium. The influence of preparative parameters like amount of the effervescent base in the tablet and its effect on the floating ability of the formulation, variables like polymer type, polymer: polymer ratio and drug: polymer ratio was also investigated. The formulation was optimized based on trial and error method.

## Results and Discussion

### Compressibility, flow property and angle of repose

The prepared tablets were evaluated for various physical properties. The bulk densities for the powders of various formulations ranged between 0.582 ± 0.42 gm/ml and 1.462 ± 0.79 gm/ml, as determined by the tap method. This value of bulk density indicates good packing character. The compressibility index (CI) for all the formulations was found to be below 17%, indicating desirable flow properties [7]. The flow properties of granules were further analyzed by determining the angle of repose for all granules; it ranged between 28.51° ± 0.72° to 31.24° ± 0.87°. The value indicates good flow property [8] of powders.

### Evaluation of post compression parameters:

The compressed tablets were evaluated for their weight variation, friability, content uniformity, and diameter. As shown in table 1, all the parameters were found to be within range.

### Drug Content

The amount of drug in the formulations F1–F6 was found to be within the range. The results are shown in table 1.

### Evaluation of buoyancy of the tablets

The *in vitro* buoyancy studies in SGF pH 1.2, revealed good buoyancy for all the formulations (Table 2). Citric acid and sodium bicarbonate combination was used as the effervescent base [9]. Upon contact with the acidic medium, the fluid permeated into the tablet, causing neutralization reaction to occur, which generates carbon dioxide (CO_2_). The swelling polymer traps the CO_2_ so generated and thus provides continued buoyancy. Preliminary studies were done to estimate the ideal amount of the effervescent base needed to obtain short floating lag time together with prolonged buoyancy. The study revealed that citric acid and sodium bicarbonate in the amount 15mg and 75 mg respectively, were optimum for the desired formulation to provide good buoyancy with floating lag time less than a minute. All the tablets floated in the buffer solution for more than 12 h except formulation F1, which showed rapid tablet disintegration on contact with the dissolution media. While formulation F4 showed the disintegration after 8 h. The gas generating base decreases the lag time by accelerating the hydration of the swelling polymer, thus allowing a higher floating duration because of constant generation and subsequent trapping of CO_2_ [10]. Citric acid was used to accelerate the CO_2_ generation; also it permits the generation of CO_2_ even if the gastric pH is abnormally high. From the preliminary trial batches it was observed that when Carbopol 934P was used in higher concentration, the floating lag time was decreased while in case of NaCMC, when the concentration was increased floating lag time was also increased.

### Percent Swelling Index

**Fig. 1.**
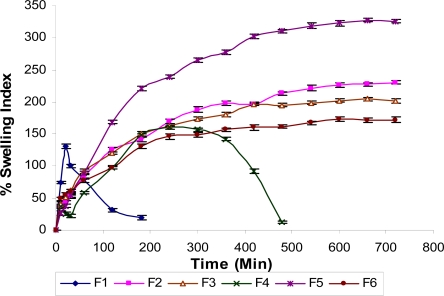
Percent swelling index profile of formulations F1–F6.

In controlled or sustained release formulations, diffusion, swelling and erosion are the three most important rate-controlling mechanisms followed. The swelling of the polymers can be measured by their ability to absorb water and swell. Swelling index (%) was studied for all the formulations and the swelling profiles of formulations F1–F6 are shown in figure 1. From the figure it can be revealed that formulations F1 and F4 disintegrated after 2 h and 8 h respectively after complete swelling. In formulation F1, amount of HPMC K15M was not enough to maintain the matrix integrity and in case of formulation F4, NaCMC was used, which gives erosion property in less amount probably because of which the tablet disintegrated after 8 h. In case of formulations F2, F3 and F6, the swelling was higher in F2 than F3 and F6, while amongst F3 and F6, swelling was more in F3. This was due to the amount of HPMC K15M. As its amount was increased, swelling of tablet increased. In formulation F5 along with HPMC K15M, Carbopol 934P was also used and due to its high swelling property maximum swelling was achieved in this formulation in comparison to other formulations.

### Effect of polymer concentration on matrix integrity of the tablets

Maintenance of matrix integrity of the tablets is an important parameter that needs to be studied in case of oral sustained release formulation. If the tablet does not maintain its physical integrity, it could be broken down into smaller fragments and escape from the upper part of the GI tract [11, 12]. In our study a highly soluble drug propranolol HCl, which is unstable in alkaline pH has been formulated into sustained release floating formulation. Our preliminary study indicated that ≤ 27.5% concentration of HPMC K15M was needed as the swelling agent in order to maintain sufficient matrix integrity of the tablets when citric acid and sodium bicarbonate were used in amount of 15 mg and 75 mg respectively, as the effervescent base and DCP as the diluents. Tablets with <27.5% concentration of HPMC K15M could not maintain matrix integrity and disintegrated soon. Formulation F2, F3, F5 and F6 maintained good matrix integrity. In case of F4, matrix integrity was not maintained upto 12 h and disintegrated completely after 8 h as well as rapid erosion of the tablet was observed when placed in contact with the dissolution medium. This was due to the higher solubility of NaCMC than HPMC [13] and perhaps due to the disintegrating effect of NaCMC. Propranolol HCl interacts with NaCMC to form a propranolol CMC precipitate. This interaction reduces the release rate of Propranolol HCl provided there was sufficient HPMC K15M to maintain the integrity of the matrix [13]. But in case of formulation F1, HPMC K15M was not sufficient enough to maintain the matrix integrity and this probably led to rapid erosion of the tablet. In case of F5, the combined effect of swelling agent HPMC K15M and carbopol 934P were responsible for the maintenance of matrix integrity.

### In vitro drug release

**Fig. 2.**
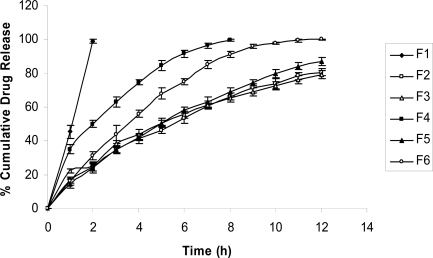
*In vitro* dissolution profile of Propranolol HCl from formulations F1–F6 (n=3 ± S.D.)

The floating lag time in all the formulations was studied at the time of dissolution also. The lag time was found to be less than a minute for all the formulations. Figure 2 shows the release profile of all six formulations in SGF pH 1.2. All the developed formulations were able to efficiently control propranolol HCl release over a time period of 12 h, except F1 and F4, where tablet erosion and disintegration was seen. When only drug and HPMC K15M was used along with the effervescent base, the hydrophilic swelling agent formed a thick gel upon hydration. This gel layer was responsible for maintenance of the matrix integrity, avoidance of burst effect and the slow release of the drug from this formulation (F6). While in case of formulation F1 amount of HPMC K15M was not sufficient to maintain the integrity and showed complete disintegration in 2 h. Formulations F2, F3 and F5 showed better drug release profile than F4. Drug release was slow from formulations F2, F3 and F5 and the release of drug from these were found to be 80.66% ± 1.9%, 79.1%± 2.3% and 87.16% ± 2.4% respectively. They failed to release the complete drug at 12 h. Here better drug release was evident due to the amount of the swelling agent and also probably due to the presence of DCP which is hydrophobic in nature, but soluble in acidic solution. The solubility of drug and DCP together with the reduction in the amount of the gelling agent probably led to an increase in the overall percentage release of propranolol HCl. Our preliminary studies have revealed that, further reduction in concentration of HPMC which was counter-balanced by an increase in the concentration of the diluent in order to maintain constant tablet weight, led to tablet disintegration as these were unable to maintain physical integrity of the tablets under the conditions of the experiment. Formulation F5 containing a combination of carbopol and HPMC was able to maintain a firm matrix, showed good water uptake and was able to sustain the drug release and only 87.16% drug was released at the end of 12 h. F4 showed rapid drug release. The tablet was not able to maintain matrix integrity and disintegrated soon. This was probably due to the disintegrant nature of NaCMC as well as due to its higher solubility than HPMC. Also, the percentage of effervescent base used (22.5%) may be responsible for this effect. All the formulations followed the non-fickian (anomalous) transport mechanism except F1. The release mechanism of formulation F1 was not studied because it was found to be disintegrated within 2 h only. Korsemeyer-Peppas model was found to be best fitted with anomalous diffusion for all the formulations with n values between 0.5 to 1 [19]. The results of various models are shown in table 3.

### FTIR study

**Fig. 3.**
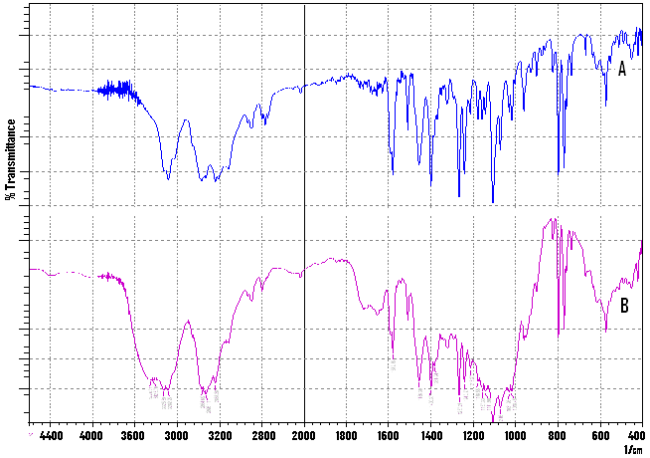
FTIR spectrum of Propranolol HCl (A) and physical mixture of Propranolol HCl with excipients used (B).

Drug polymer interaction was checked by comparing the IR spectra of the physical mixture of drug with the excipients used with the IR spectrum of pure drug. As shown in Figure 3 (A), Propranolol HCl gives the peaks in IR spectrum nearby at 2965 cm^−1^ due to the presence of a secondary amine group, 3283 cm^−1^ due to the hydroxyl group (secondary), the aryl alkyl ether display a stretching band at 1267.27 cm^−1^ and the peak at 798 cm^−1^ due to a-substituted naphthalene [15]. Figure 3 (B) revealed the presence of peaks at 2964.69 cm^−1^, 3282.95 cm^−1^, 1267.27 cm^−1^ and 798.2 cm^−1^. Frequencies of functional groups of pure drug remained intact in physical mixture containing different polymers; hence, there was no major interaction between the drug and excipients used in the study. This established the stability of the drug in the formulation which was further confirmed by Differential Scanning Calorimetry (DSC) thermograms.

### Differential Scanning Calorimetry (DSC)

**Fig. 5.**
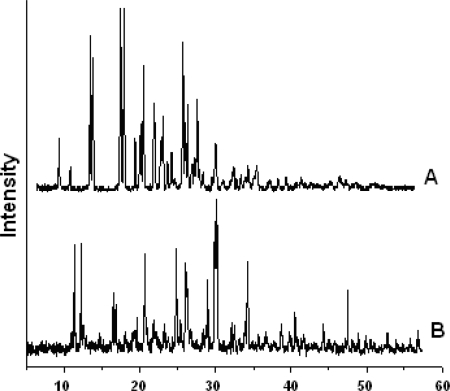
XRPD spectrum of Propranolol hydrochloride (A) and Formulation F6 (B).

Thermograms of the pure drug and the formulation F6 is shown in figure 5. A sharp melting transition of propranolol hydrochloride pure drug was observed at 166.193°C. In formulation F6 melting endotherm at 164.074°C was observed. This confirmed that the presence of other excipients did not affect the drug nature and it was well maintained in the selected formulation [16].

### X-Ray Powder Diffraction (XRPD)

The X-ray powder diffraction spectra of pure Propranolol HCl and Formulation F6 (Figure 5) were measured for comparison. Pure Propranolol HCl showed a typical pattern of crystalline substance which showed characteristic sharp refraction peaks in the range of 2θ scattered. The sharp peaks of drug were also present in formulation F6, where it was mixed with the polymers. This indicated that the crystalline nature of the drug formulation was not disturbed even after formulation [16–18].

## Experimental

### Materials

Propranolol hydrochloride was obtained as a gift sample from Alkem pharma Ltd., Mumbai, hydroxypropyl methylcellulose (HPMC K15M) was kindly supplied as gift sample from Colorcon, Goa. Carbopol 934P was gifted by Corel Pharma-Chem, Ahmedabad, sodium bicarbonate was obtained from Central Drug House (P) Ltd., New Delhi, dibasic calcium phosphate (DCP) was obtained from Rankem, New Delhi. Sodium carboxymethyl cellulose (NaCMC), magnesium stearate and talc were purchased from SD fine chemicals, Mumbai.

### Methods

#### Preparation of tablets

Direct compression method was used to prepare the floating tablets. Various preliminary trial batches were prepared to get desired concentration of ingredients. Compositions of each tablet for each batch are shown in table 4. All the excipients were passed through sieve no. 72, and mixed thoroughly for 10 min by geometrical mixing. Each of the tablet formulation consisted of 80 mg propranolol hydrochloride, 15 mg citric acid and 75 mg sodium bicarbonate (NaHCO_3_). Magnesium stearate and talc, 2mg each, were used as lubricant and glidant respectively. The ratio of citric acid to NaHCO_3_ was kept as 1:5 [20]. The amount of HPMC K15M was varied for various formulations along with NaCMC and Carbopol 934P. The amounts of NaCMC and Carbopol 934P in combinations with HPMC K15M were optimized based on floating lag time, floating time and release profile after various preliminary trial batches. The total weight of a tablet was kept 400mg and these were punched using 11 mm punches. HPMC offers the advantages of being non-toxic and relatively inexpensive and also it can be compressed directly into matrices, so it was used in this study [6].

Prior to compression, powders were evaluated for their characteristic parameters, such as bulk density, tapped density, Carr’s index and angle of repose [8]. Carr’s compressibility index was calculated from the bulk and tapped densities [7] using a tap density apparatus (Galaxy scientific equipments, India).

#### Post compression parameters

The tablets were evaluated for their hardness, thickness, weight variation and friability. The results are shown in table 1.

#### Drug content

Ten tablets were weighed and powdered, 400 mg from it was transferred to a 100 ml volumetric flask, 5 ml of dilute HCl was added, and it was allowed to stand and swirled occasionally. About 70 ml of methanol was added, shaken well and the volume was made up. It was mixed and a small portion of the solution was centrifuged. A suitable volume of this was then diluted with methanol to obtain a solution containing 40 μg of propranolol hydrochloride per ml. The extinction of 1 cm layer of the resulting solution was measured at a maximum of about 290 nm using methanol as the blank solution.

#### Buoyancy lag time determination

The buoyancy of the tablets was studied at 37 ± 0.5°C, in 100 ml of Simulated Gastric Fluid (SGF), pH 1.2 (USP XXIII). The floating lag time and floating durations were noticed [20].

#### Swelling Index

The studies were carried out in petridishes using Hydrochloric acid buffer (pH 1.2); the prepared tablets were introduced in swelling media along with the preweighed OHP sheet, placing the tablet on it. At predetermined time intervals the tablets were removed from medium, the excess fluid was blotted with tissue paper and immediately weighed. The swelling index was calculated using following formula,

$$\%\;\text{Swelling}\;\text{Index}\;=\;{\text{W}}_{\text{o}}-\left(\frac{{\text{W}}_{\text{i}}{+\text{W}}_{\text{b}}}{{\text{W}}_{\text{i}}}\right)\times 100$$

Where,

W_o_ = Weight of swollen tablet along with OHP sheet
W_i_ = Weight of initial tablet
W_b_ = Weight of OHP paper.

#### In vitro dissolution test

The release of Propranolol hydrochloride from the tablet was studied using USP XXIII paddle apparatus (Electrolab, Bangalore). Drug release profile was carried out in 900 ml of SGF maintained at 37 ± 0.5°C temperature at 100 rpm. Ten ml of samples were withdrawn at predetermined time intervals of every 1 h up to 12 h. The samples were replaced by its equivalent volume of dissolution medium and were filtered through 0.45 μm Whatman filter paper and assayed at 290 nm by UV spectrophotometer (Shimadzu 1601, Japan).

#### Mechanism of the in vitro release

The drug release data were evaluated by the model-dependent (curve fitting) method. In the present study, the Korsmeyer-Peppas model describing drug release from polymeric system was used. This model takes into account that the drug release mechanism often deviates from the Fick’s law and follows anomalous behavior described by the following equation [14]:

Eq. 1. $$\frac{M_{t}}{M_{\propto }}=\text{k}\cdot {\text{t}}^{\text{n}}$$

Where, M_t_ is the drug released at time t, M_∞_ the quantity of drug released at infinite time, k the kinetic constant and n is the release exponent. The value of n is related to the geometrical shape of the delivery systems and determines the release mechanism.

The release data was further treated according to Higuchi equation:

Eq. 2. $$\text{Q}=\text{k}\cdot {\text{t}}^{\frac{1}{2}}$$

Where, Q is the percent of drug released at time t and k is the kinetic constant.

The value of n in equation (1) determines the mechanism of drug release. When n approximates to 0.5, a Fickian/diffusion controlled release is implied, where 0.5<n<1.0 non-Fickian transport and for n=1 zero order (case II transport). When n approaches 1.0, phenomenologically one may conclude that the release is approaching zero order [19].

#### Fourier transform infrared spectroscopy (FTIR study)

Infrared spectrum was taken (FT-IR, 8400S, Shimadzu) by scanning the sample in potassium bromide discs. The samples of pure drug and physical mixture containing different polymers and excipients were scanned individually.

#### Differential Scanning Calorimetry (DSC)

DSC studies were carried out using Perkin Elmer DSC-7 instrument. Samples were sealed hermetically in flat bottom aluminium cells. These were then heated over a temperature range of 50–205°C in an atmosphere of nitrogen (20 ml/min) at a constant rate of 10°C/min.

#### X-ray powder diffractometry (XRPD)

XRPD of pure drug and the selected formulation (F6) was carried out and compared to check the nature of the drug in the formulation. Philips X’Pert PW 3040/60 (Almelo, Netherlands) was used as X-ray generator for Cu Kα radiation (λ = 1.54178 Å). Data was collected in the continuous scan mode using step size of 0.01° 2θ. The scanned range was 5–50°.

## Conclusion

In the present work floating tablets have been prepared incorporating a highly soluble antihypertensive drug propranolol HCl. Gas formation technique was used to keep the tablets floating over the SGF pH 1.2 for more than 12 h. All the formulations showed floating lag time less than a min. Mixture of citric acid and sodium bicarbonate was found to be a good effervescent base. A non-Fickian diffusion was confirmed as the drug release mechanism for these formulations. This meant that water diffusion and polymer rearrangement have essential roles in the drug release. Tablets containing 27.5% HPMC K15M, 29% DCP, 3.75% citric acid and 18.75% sodium bicarbonate were optimum from both duration of buoyancy and drug release point of view and were found to be stable under the stability conditions. Thus floating drug delivery system provides a method for sustaining the drug release of a highly soluble drug propranolol HCl in stomach.

## Authors’ Statement

### Competing Interests

The authors declare no conflict of interest.

## Figures and Tables

**Fig. 4.**
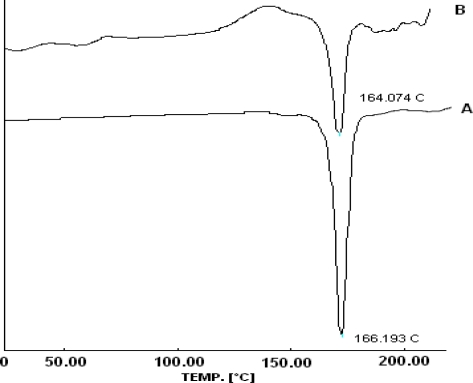
DSC spectrum of (A) Propranolol HCl and (B) formulation F6.

**Tab. 1.** Post compression parameters for formulations F1–F6.

**Formulation Code**	**Friability (%)**	**Thickness (n=20 ± SD) (mm)**	**Hardness^#^ (n=10 ± SD) (Kg/cm^2^)**	Weight* (n=20 ± SD) (mg)	**Drug Content (%)**
F1	0.2	4.73 ± 0.1	4.68 ± 0.2	401.4 ± 2.9	97.82
F2	0.2	4.66 ± 0.1	4.73 ± 0.1	399.6 ± 2.7	102.16
F3	0.4	4.70 ± 0.2	4.53 ± 0.3	400.6 ± 2.4	104.26
F4	0.5	4.53 ± 0.1	4.83 ± 0.3	402.2 ± 4.0	95.48
F5	0.3	4.46 ± 0.2	4.40 ± 0.4	401.0 ± 3.4	99.35
F6	0.2	4.33 ± 0.2	4.86 ± 0.3	400.8 ± 3.6	101.33

**Tab. 2.** Buoyancy study for formulations F1–F6 (n=3 ± S.D)

**Formulation code**	**Floating lag time (sec)**	**Floating duration (h)**
F1	12 ± 1.4	Disintegrated after 2 h
F2	15 ± 3.6	More than 12 h
F3	20 ± 2.9	More than 12 h
F4	22 ± 3.8	Disintegrated after 8 h
F5	11 ± 4.2	More than 12 h
F6	13 ± 2.7	More than 12 h

**Tab. 3.** Model fitting for the formulations F1–F6.

**Sl. No**	**Formulation**	**Zero order**	**First order**	**Higuchi**	**Korsemeyer-Peppas**	

		**R^2^**	**R^2^**	**R^2^**	**n**	**R^2^**
1	F1	–	–	–	–	–
2	F2	0.9613	0.9968	0.9869	0.6528	0.9967
3	F3	0.9357	0.9960	0.9932	0.5559	0.9793
4	F4	0.9050	0.8394	0.9963	0.5201	0.9952
5	F5	0.7999	0.9534	0.9190	0.7367	0.8914
6	F6	0.9697	0.9879	0.9812	0.7290	0.9966

**Tab. 4.** Composition of the floating tablets prepared*

**Ingredients (mg/tablet)**	**Formulations**					
	**F1**	**F2**	**F3**	**F4**	**F5**	**F6**
Propranolol HCl	80	80	80	80	80	80
HPMC K15M	85	185	125	100	140	110
DCP	141	41	101	106	76	116
NaCMC	–	–	–	20	–	–
Carbopol 934P	–	–	–	–	10	–

* All tablets contain citric acid and sodium bicarbonate 15 mg and 75 mg respectively and talc and magnesium stearate 2 mg each. Tablet weight was maintained as 400 mg.
